# The Role and Mechanism of TRIM Proteins in Gastric Cancer

**DOI:** 10.3390/cells13242107

**Published:** 2024-12-19

**Authors:** Wangxi Wu, Jinyu Yang, Tian Yu, Zhuoling Zou, Xuan Huang

**Affiliations:** 1 The National Engineering Research Center for Bioengineering Drugs and the Technologies, Jiangxi Provincial Key Laboratory of Bioengineering Drugs, Institute of Translational Medicine, Jiangxi Medical College, Nanchang University, Nanchang 330031, China; wangxi.wu@se21.qmul.ac.uk (W.W.); ytian076@163.com (T.Y.); 2 The Queen Mary School, Jiangxi Medical College, Nanchang University, Nanchang 330031, China; 4217121019@email.ncu.edu.cn (J.Y.); 4217120140@email.ncu.edu.cn (Z.Z.); 3 Chongqing Research Institute, Nanchang University, Chongqing 400010, China

**Keywords:** TRIM proteins, E3 ubiquitin ligase, ubiquitylation, gastric cancer, signaling pathways

## Abstract

Tripartite motif (TRIM) family proteins, distinguished by their N-terminal region that includes a Really Interesting New Gene (RING) domain with E3 ligase activity, two B-box domains, and a coiled-coil region, have been recognized as significant contributors in carcinogenesis, primarily via the ubiquitin–proteasome system (UPS) for degrading proteins. Mechanistically, these proteins modulate a variety of signaling pathways, including Wnt/β-catenin, PI3K/AKT, and TGF-β/Smad, contributing to cellular regulation, and also impact cellular activities through non-signaling mechanisms, including modulation of gene transcription, protein degradation, and stability via protein–protein interactions. Currently, growing evidence indicates that TRIM proteins emerge as potential regulators in gastric cancer, exhibiting both tumor-suppressive and oncogenic roles. Given their critical involvement in cellular processes and the notable challenges of gastric cancer, exploring the specific contributions of TRIM proteins to this disease is necessary. Consequently, this review elucidates the roles and mechanisms of TRIM proteins in gastric cancer, emphasizing their potential as therapeutic targets and prognostic factors.

## 1. Introduction

Gastric cancer (GC), ranked fifth globally with regard to both incidence and mortality, is still a serious health concern. This reflects the underlying complexity of gastric cancer, which presents a significant challenge to healthcare systems [1]. Gastric cancer develops through a multistep process described by Correa’s cascade, progressing from chronic gastritis to atrophic gastritis, intestinal metaplasia, dysplasia, and ultimately adenocarcinoma [2]. This progression is mainly driven by Helicobacter pylori infection and other factors, including high-salt diets, smoking, and genetic mutations such as CDH1 [3].

The prognosis for patients with gastric cancer is poor, as demonstrated by the 5-year survival rate of only 33%, which is attributed to the biological and clinical challenges posed by the disease [4]. Despite some advances in treatment, many characteristics of gastric cancer, including high rates of metastasis and recurrence, late-stage diagnosis, and chemoresistance, often restrict the effectiveness of conventional therapies such as surgery and chemotherapy [5]. Metastasis is the primary factor contributing to the unfavorable prognosis of gastric cancer [6]. Once established, gastric cancer commonly metastasizes to the liver, peritoneum, lungs, and bones, following diverse routes of dissemination, including distant spread through the hematogenous and lymphatic systems or direct invasion [7]. Key drivers of these processes include the epithelial–mesenchymal transition (EMT), matrix metalloproteinases (MMPs), and tumor–stromal interactions. EMT promotes cancer cell motility by reducing cell adhesion, while MMPs degrade the extracellular matrix and basement membrane, enabling tissue invasion and dissemination [6,8,9]. Meanwhile, stromal cells like tumor-associated fibroblasts (CAFs) and mesenchymal stem cells (MSCs) secrete pro-inflammatory cytokines and growth factors, enhancing the invasive and migratory abilities of cancer cells [6,10].

Dysfunction of the ubiquitin–proteasome system (UPS), a key mechanism for protein degradation, has been observed in gastric cancer development. Target proteins are ubiquitinated by the UPS, causing their degradation via the proteasome-mediated pathway [11]. In gastric cancer, protein accumulation is caused by the abnormal alteration of enzymes that are essential for the UPS, including ubiquitin-activating enzyme (E1), ubiquitin-conjugating enzyme (E2), and ubiquitin ligase (E3) [12]. During the early stages of this process, ubiquitin is activated and linked to E1 via a thiol–ester bond, which is then similarly transferred to E2. Subsequently, E3 ubiquitin ligases, which are classified as RING–IBR–RING (RBR) family, homologous to the E6–AP COOH terminus (HECT) family, U-box family, and Really Interesting New Gene (RING) family, the primary group of E3 ligases, perform functions through distinct mechanisms [13,14]. In particular, RING E3 ligases connect to the E2 loaded with ubiquitin, bringing the substrate into proximity for direct ubiquitin transfer [15,16]. The effect on substrate degradation or modification is contingent upon the type of ubiquitinated chain to which the lysine (K) is bound. For instance, protease-mediated protein degradation is attributed to K48-linked chains, while K63-linked chains mediate DNA repair and regulate the activity of protein kinase [17].

With most of them displaying E3 ligase activity, Tripartite motif (TRIM) family proteins are becoming a subject of intense research interest. Currently, there are over 80 known TRIM family proteins, with the majority featuring an N-terminal region comprising a RING domain, two B-box domains, and a coiled-coil region [18,19]. The E3 ubiquitin ligase activity, necessary for substrate recognition in ubiquitination, is mediated by the RING domain [17,20]. Based on their ubiquitination activity, TRIM proteins regulate several vital cellular processes, including transcription, intracellular signaling, innate immunity, and cancer development [21]. Furthermore, mounting evidence indicates that TRIM proteins exhibit dual roles in cancer, either promoting or inhibiting oncogenesis and tumor progression by influencing pathways related to DNA damage repair, cell growth, and apoptosis [21,22].

This review delves into the role of TRIM proteins in gastric cancer, examining how these proteins influence tumor progression through both signaling pathways and non-signaling mechanisms. Furthermore, their potential as prognostic factors and therapeutic targets is also explored, highlighting their clinical significance.

## 2. The Structure and Functions of TRIM Family Protein Domains

**Figure 1:**
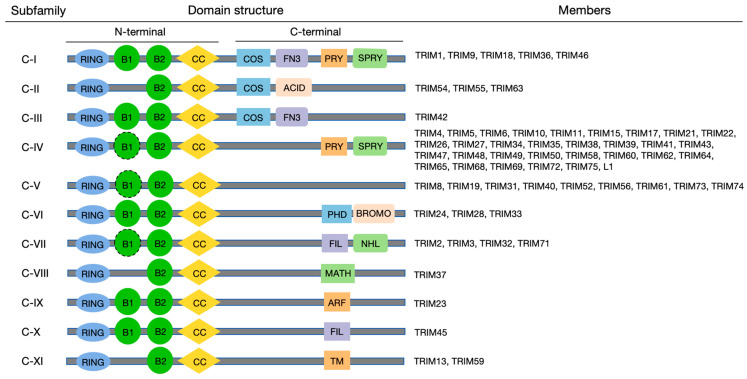
Structure of TRIM proteins. TRIM proteins are grouped into 11 subfamilies, from C–I to C–XI. List of abbreviations: B1 (B-box domain), B2 (B-box domain), CC (coiled-coil domain), COS (cos-box domain), FN3 (fibronectin type III domain), PRY-SPRY (PRY-SPRY domain/B30.2), ACID (additional acid-rich region domain), PHD (plant homeodomain domain), BROMO (bromodomain domain), FIL (filamin-type immunoglobulin domain), NHL (NCL-1/HT2A/LIN-41 domain), MATH (meprin and TRAF-homology domain), ARF (ADP-ribosylation factor domain), TM (transmembrane domain).

TRIM proteins belong to the RING family of E3 ubiquitin ligases, featuring an N-terminal domain consisting of an RBCC (RING finger/B-box/coiled-coil) motif, along with a C-terminal domain that has distinct activities (Figure 1) [23,24].

### 2.1. The N-Terminal Region

TRIM proteins are characterized by their RBCC motifs, which encompass RING, B-box, and coiled-coil domains. These domains perform distinct modular functions within the TRIM protein structure [25,26].

In general, the RING domain features a motif with cysteine and histidine that facilitates the binding of two zinc atoms, making it an essential domain for ubiquitination [17,27]. Meanwhile, this domain is present in many proteins with varied activities [26]. The RING domain facilitates the ubiquitin transfer by engaging the substrate and the E2 enzyme. This is achieved by creating an isopeptide bond between the lysine residue of the substrate and the ubiquitin C-terminal, which results in the substrate’s distinct outcomes [28,29].

The B-box domains that come after the RING domain also have a zinc-binding motif, the integrity of which is crucial for the normal activity of TRIM proteins [25,27]. Most TRIM proteins contain two distinct B-boxes, B-Box1 and B-Box2, which differ in sequence and number. B-Box1 is typically positioned closer to the N-terminal than B-Box2 when both are present. Notably, B-Box1 contains a cysteine, while B-Box2 features a histidine [20,30]. Recent studies have indicated the critical role of B-box1 in the TRIM protein family, as it promotes the E3 ligase function of the RING domain [31,32].

The coiled-coil domain follows the B-box domain, which is composed of many intertwining α-helices, with lengths varying from 30 to 110 residues among TRIM proteins [17,32]. The coiled-coil domain facilitates the large complex’s formation and the execution of diverse biological functions in TRIM proteins. Homodimerization and heterodimerization are two main modes of interaction, with the former being more prevalent, suggesting it has higher specificity than heterodimerization [17,20,32].

### 2.2. The C-Terminal Region

The C-terminal domain of TRIM proteins varies greatly, contributing to their diverse properties [19,33]. With different domain combinations, more than eighty identified TRIM proteins are categorized into eleven subfamilies (C–I to C–XI) and an unclassified group lacking the RING finger domain [34,35].

The PRY-SPRY domain, also referred to as B30.2, is the most common domain at the C-terminals of TRIM proteins. It facilitates protein interaction, as exemplified by TRIM14, which plays a role in combating pathogens [36,37]. The COS (cos-box) domain, situated near the N-terminal of TRIM proteins, is essential for maintaining microtubule association. For instance, TRIM9 has been demonstrated to interact with the cytoskeleton [38,39]. The FN3 (fibronectin type III repeat) domain is frequently situated subsequent to the COS domain, except for TRIM76. It is invariably present in cell surface proteins, where it performs a molecular recognition function. Furthermore, the ACID (additional acid-rich region) domain, situated subsequent to the COS domain in the C–II family, regulates muscle protein turnover via the ubiquitin–proteasome pathway [40]. The PHD (plant homeodomain) domain is involved in chromatin-mediated transcriptional modulation, cellular development, differentiation, and homeostasis [41,42]. The NHL (NCL-I/HT2A/LIN-41 repeat) domain, derived from the NCL-1/HT2A/LIN-41 protein, is characteristic of the C–VII family and essential for protein binding, as seen in TRIM32 and p53 [43,44]. Moreover, the NHL domain is situated at the C-terminal end of the TRIM protein, while the FIL (filamin-type immunoglobulin domain) domain is frequently located adjacent to it and participates in the regulation of mRNA [33]. The MATH (meprin and TRAF-homology) domain is specific to the C–VIII family, which reacts with tumor necrosis factor (TNF) receptors and is important in receptor interactions associated with TNF receptor-associated factor (TRAF) [45]. The ARF (ADP-ribosylation factor) domain, which is specific to the C–IX family, is known to interact with TRIM23. This interaction is necessary for TRIM23-mediated autophagy, which occurs through an unconventional K27-linked auto-ubiquitination process [46]. In addition, the C–XI family’s C-terminal region contains a distinct TM (transmembrane) domain [47]. Notably, the C–V family is involved in regulating inflammatory pathways and lacks a C-terminal domain [48,49,50].

To sum up, the specific properties and different functions of TRIM proteins are defined by the variable C-terminal region.

## 3. Role of TRIM Proteins in Signaling Pathways in Gastric Cancer Development

**Figure 2:**
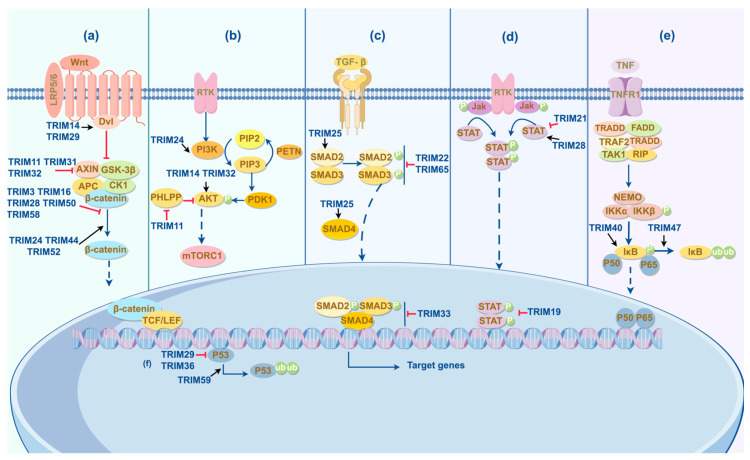
TRIM proteins in the signaling pathways. TRIM proteins play roles in (**a**) the Wnt/β-catenin signaling pathway, (**b**) the PI3K/AKT signaling pathway, (**c**) the TGF-β/Smad signaling pathway, (**d**) the JAK-STAT signaling pathway, (**e**) the NF-κB signaling pathway, and (**f**) the P53 signaling pathway. Arrows with a perpendicular line at the tail: inhibit; arrows: promote. List of abbreviations: LRP5/LRP6 (low-density-lipoprotein-related protein 5/6), Dvl (Dishevelled), GSK-3β (glycogen synthase kinase 3 beta), APC (adenomatous polyposis coli), CK1 (casein kinase 1), TCF/LEF (T cell-specific factor/lymphoid enhancer-binding factor), RTK (receptor tyrosine kinase), PI3K (phosphoinositide 3-kinase), PIP2 (phosphatidylinositol diphosphate), PIP3 (phosphatidylinositol 3-phosphate), PTEN (phosphatase and tensin homolog), AKT (Protein Kinase B), PHLPP (pleckstrin homology domain leucine-rich repeat protein phosphatase), PDK1 (phosphoinositide-dependent protein kinase-1), mTORC1 (mechanistic target of rapamycin complex 1), TGF-β (transforming growth factor-β), SMAD (small mothers against decapentaplegic), Jak (janus kinase), STAT (signal transducer and activator of transcription), TNF (tumor necrosis factor), TNFR1 (tumor necrosis factor receptor 1), TRADD (tumor necrosis factor receptor type 1-associated death domain protein), FADD (Fas-associated protein with death domain), TRAF2 (TNF receptor-associated factor 2), TAK1 (transforming growth factor-beta-activated kinase 1), RIP (receptor-interacting protein), NEMO (NF-κB essential modulator), IκB (inhibitor of κB), IKK (IκB kinase).

In gastric cancer development, multiple major signaling pathways, including EGFR/HER2, p53, PI3K/AKT, MAPK, TGF-β, Wnt/β-catenin, Notch, JAK/STAT, NF-κB, and Hedgehog, have been identified as essential for regulating apoptosis, cell cycle, proliferation, migration, and invasion [51]. Notably, recent research has revealed potential interactions between TRIM protein expression and functional regulation of the Wnt/β-catenin, PI3K/AKT, and TGF-β pathways [52]. Given the critical roles of TRIM proteins in disease progression and the frequent alterations of these pathways in gastric cancer, we primarily focus on how TRIM proteins impact gastric cancer progression through these three pathways while also considering other, less extensively studied ones (Figure 2). The effects and mechanisms of TRIM proteins in gastric cancer are shown below.

### 3.1. Wnt/β-Catenin Signaling Pathway

#### 3.1.1. Overall Role and Mechanism of Wnt/β-Catenin Signaling Pathway

The canonical Wnt signaling pathway, also known as the Wnt/β-catenin signaling pathway, plays roles in various cellular activities relevant to tumors, including invasion, proliferation, migration, and differentiation [53,54,55]. The highly conserved Wnt protein functions as a growth stimulatory factor to promote cell proliferation and as a directional growth factor to induce cell differentiation [56,57,58,59]. During the signaling process, when the Wnt/β-catenin pathway is inactivated, the degradation complex, including adenomatous polyposis coli (APC), AXIN, casein kinase 1 (CK1), and glycogen synthase kinase 3 protein (GSK3), phosphorylates β-catenin. This phosphorylation induces β-catenin to be ubiquitinated and then degraded, which decreases cytoplasmic β-catenin levels [60,61,62]. Conversely, upon the activation of the Wnt/β-catenin pathway, Wnt ligands attach to the transmembrane receptor, Frizzled (FZD), and low-density-lipoprotein-related protein 5/6 (LRP5/LRP6), and then Dishevelled (DVL) in the cytoplasm associates with FZD, recruiting the degradation complex to the receptor [60,63]. GSK3β is then phosphorylated and inhibited, rendering the degradation complex inactive, resulting in increased levels of cytoplasmic β-catenin [64]. After migrating to the nucleus, β-catenin binds to T cell-specific factor (TCF)/lymphoid enhancer-binding factor (LEF), triggering the transcription of target genes, which accelerates the growth of tumors, like *cyclin D1* and *c-myc* [65,66,67,68].

#### 3.1.2. TRIM Proteins Act as Activators of the Wnt/β-Catenin Signaling Pathway

Recently, some studies have revealed that TRIM proteins can function as activators in the Wnt/β-catenin signaling pathway.

TRIM11 was upregulated in gastric cancer tissues of patients. In vitro experiments using human gastric cancer cells HGC-27 demonstrated that TRIM11 overexpression enhanced cell proliferation, migration, and invasion, while its knockdown had the opposite effects. In vivo, TRIM11 knockdown significantly reduced tumor growth in a subcutaneous xenograft model using BALB/c nude mice, highlighting its oncogenic role in gastric cancer progression via activation of the β-catenin signaling pathway [69]. Mechanistically, another study indicated that TRIM11 can destabilize Axin1 and promote its degradation via ubiquitination, resulting in the β-catenin pathway activation in gastric cancer cells and lymphoma cells [67,70]. Collectively, these findings underscore that TRIM11 contributes to gastric cancer progression by strengthening β-catenin activity through targeted degradation of Axin1, thereby driving tumor growth and invasiveness. TRIM14 was overexpressed in gastric cancer tissues and OXA-resistant human gastric cancer cells, HGC-27/OXA. TRIM14 knockdown in HGC-27/OXA cells accelerated Dvl2 degradation, reducing autophagy and OXA resistance. In vivo, orthotopic implantation of HGC-27/OXA cells into SCID-Beige mice showed that TRIM14 promoted tumor growth. This suggested that TRIM14 maintained Dvl2 stability to activate the Wnt/β-catenin signaling pathway, thereby promoting OXA resistance and autophagy in cancer cells, highlighting its potential as a target to overcome drug resistance. In addition, in vitro studies have demonstrated that circ_0091741 functions by competitively binding to miR-330-3p, thereby suppressing its activity and alleviating its regulatory suppression of TRIM14, ultimately leading to enhanced TRIM14 expression [71]. TRIM24 was overexpressed in gastric cancer tissues and cell lines (MGC803 and HGC-27), with a positive correlation observed between the mRNA levels of TRIM24 and β-catenin. TRIM24 silencing in MGC803 and HGC-27 cells led to decreased Wnt/β-catenin target gene expression, whereas activating the Wnt/β-catenin pathway reversed this effect, which suggested that TRIM24 can positively regulate this pathway [72]. Additionally, in gastric cancer, miR-511 had a decreased expression level in human gastric cancer tissues and cell lines (MGC803 and HGC-27), which was negatively associated with TRIM24. Overexpression of miR-511 in MGC803 and HGC-27 cells resulted in the downregulation of TRIM24 expression, thus suppressing the Wnt/β-catenin pathway and inhibiting gastric cancer progression [73]. Overall, TRIM24 emerges as a key modulator connecting β-catenin signaling with microRNA networks, triggering the Wnt pathway and promoting tumorigenesis.

TRIM29 was upregulated in human gastric cancer tissues. Its expression was positively associated with β-catenin levels, potentially leading to a worse prognosis in gastric cancer patients [74]. Knockdown of TRIM29 in gastric cancer cells SGC-7901 and MGC80-3 led to decreased β-catenin levels, as shown in vitro through siRNA-mediated experiments. In vivo, TRIM29 knockdown in a subcutaneous xenograft model using BALB/c nude mice also reduced β-catenin levels and suppressed tumor growth. This suggests that TRIM29 may play its oncogenic role through the Wnt/β-catenin signaling pathway. Moreover, overexpressed miR-185 was shown to inhibit TRIM29 at both the transcriptional and protein levels in MGC803 cells [75,76] Additionally, TRIM29 was discovered to increase Dvl2 stability in pancreatic cancer, thus leading to increased β-catenin to trigger the Wnt/β-catenin pathway, as shown by in vitro experiments in human pancreatic cancer cells Panc1 and BxPC3 and in vivo using a subcutaneous xenograft model in NOD/SCID mice [77]. Taken together, TRIM29 enhances β-catenin signaling, contributing to poor outcomes. It also interacts with regulatory elements, such as specific microRNAs and Dvl2 stabilization, making it a potential molecular target.

With increased expression in human gastric cancer tissues and cell lines (BGC-823 and HGC-27), TRIM31 was validated to ubiquitinate Axin1 for degradation to activate the Wnt/β-catenin pathway. In vitro, TRIM31 knockdown in these cell lines reduced Axin1 ubiquitination and stabilized its protein levels. In vivo, a subcutaneous xenograft model using BALB/c nude mice confirmed that TRIM31 facilitated Axin1 degradation to promote tumor growth and progression [65]. Similarly, TRIM32 was overexpressed in human gastric cancer tissues and cell lines (SGC7901 and AGS), correlating with increased levels of β-catenin. Its suppression inhibited tumor growth in vivo using a subcutaneous xenograft model in BALB/c nude mice, as well as the invasion, migration, and proliferation of gastric cancer cells SGC7901 and AGS [78]. Mechanistically, TRIM32 targets Axin1 for ubiquitination and degradation to trigger β-catenin signaling, which was demonstrated in vitro using rat nucleus pulposus cells treated with inflammatory cytokines (IL-1β or TNF-α) [79]. Altogether, TRIM31 and TRIM32 both promote cancer progression by destabilizing Axin1, leading to β-catenin activation. Furthermore, TRIM44 was found to be upregulated in human gastric cancer patients either. Analysis of the clinical samples showed that it was linked to poor overall survival (OS), and its expression level positively correlated with β-catenin expression in tumor tissues [80]. Another study revealed that TRIM44 can bind to protein 14-3-3ζ, which is critical for the stability of β-catenin, and reduce its ubiquitination at the K48 linkage to stabilize β-catenin, thus activating the Wnt/β-catenin pathway. This effect was demonstrated in vitro using human gastric cancer cells MKN45 and AGS, where TRIM44 knockdown reduced β-catenin levels and inhibited Wnt/β-catenin pathway activation [81]. Accordingly, TRIM44 serves as a stabilizer of β-catenin through 14-3-3ζ interactions, activating Wnt signaling. In Zheng et al.’s research, TRIM52 had an increased level in human gastric cancer tissues and cell lines (HGC-27, AGS, and NCI-N87). In vitro, its overexpression can promote the expression of β-catenin, c-Myc, and Wnt5a proteins, while its downregulation has the opposite effect, suggesting that it positively regulates the Wnt/β-catenin signaling pathway [82].

#### 3.1.3. TRIM Proteins Act as Inhibitors of the Wnt/β-Catenin Signaling Pathway

Meanwhile, it has been demonstrated that certain TRIM proteins block the Wnt/β-catenin signaling pathway.

Interestingly, despite increased levels of TRIM3 and TRIM16 in human gastric cancer tissues, they showed a negative correlation with the β-catenin levels and some of its downstream targets in vitro, such as *BCL2*, suggesting their tumor-suppressing function [83,84]. Notably, a contrasting conclusion about TRIM3 is presented by another study, indicating that mRNA and protein levels of TRIM3 were decreased in clinical gastric cancer tissues [85]. These findings suggest that the expression level of TRIM3 in gastric cancer remains unclear and requires further investigation. TRIM28 also showed a functional contradiction. Although TRIM28 had an increased level in human gastric cancer tissues, it was associated with improved prognosis in clinical sample analysis. TRIM28 knockdown in gastric cancer cell lines AGS and HGC-27 promoted cell stemness through activation of β-catenin signaling. Downregulated TRIM28 correlated with increased β-catenin and its downstream targets, while these impacts can be reversed by inhibitors of the Wnt/β-catenin pathway [86].

TRIM50 was downregulated in human gastric cancer tissues. In vitro, its overexpression in AGS and SGC-7901 cells suppressed cell metastasis and proliferation by facilitating the β-catenin degradation. In vivo, TRIM50 overexpression inhibited tumor growth in a subcutaneous xenograft model, while its knockdown showed the opposite effect. These changes could be reversed by inhibitors of the Wnt pathway. This demonstrates that TRIM50 enhances tumor aggressiveness by directly increasing β-catenin levels, activating the Wnt/β-catenin pathway [87]. In gastric cancer, TRIM58 was downregulated in patient tissues and cell lines (AGS and HGC27), and cellular experiments indicated that its downregulation had a negative correlation with elevated oncogenic proteins like β-catenin. On the contrary, a subcutaneous xenograft model in BALB/c nude mice confirmed that overexpression of TRIM58 can suppress these protein levels and inhibit cancer cell proliferation, suggesting that TRIM58 exerted its tumor suppressive function through β-catenin ubiquitination and degradation to inhibit β-catenin signaling [88].

In conclusion, TRIM proteins regulate essential components in the Wnt/β-catenin signaling pathway, altering its downstream target gene expression and consequently affecting gastric cancer development [70].

### 3.2. PI3K/AKT Signaling Pathway

#### 3.2.1. Overall Role and Mechanism of the PI3K/AKT Signaling Pathway

In most gastric cancer cases, the PI3K/AKT signaling pathway is dysregulated, as *PIK3CA* amplification is found in 74% of gastric cancer, leading to elevated levels of downstream molecules, such as AKT [89,90]. The significance of this pathway is manifested in various aspects such as survival, apoptosis, metastasis, angiogenesis, and tumor growth [91,92]. The critical element of the pathway, phosphoinositide 3-kinases (PI3K), consists of a regulatory subunit (p85) and a catalytic subunit (p110) [93,94]. When PI3K is activated by ligand binding to oncogenic receptor tyrosine kinases (RTKs), like epidermal growth factor receptors (EGFRs), phosphatidylinositol diphosphate (PIP2) is converted to phosphatidylinositol 3-phosphate (PIP3), while phosphatase and tensin homolog (PTEN) causes the opposite reaction [95,96,97]. Subsequently, PIP3 recruits cytoplasmic protein kinase B (AKT) and phosphoinositide-dependent protein kinase-1 (PDK1) to the cell membrane [92]. After translocating, AKT is phosphorylated by binding to PDK1 [92,98]. As a result, a variety of downstream targets of AKT are activated, such as mechanistic target of rapamycin complex 1 (mTORC1) and GSK3β [92,99,100].

#### 3.2.2. TRIM Proteins Act as Activators of the PI3K/AKT Signaling Pathway

Recent research has demonstrated that some TRIM proteins positively regulate this signaling pathway to affect gastric cancer development.

Upon TRIM11 silencing in gastric cancer cells AGS and SGC-7901, both EGFR and AKT showed decreased expression levels, indicating that it plays a role in the activation of the AKT signaling pathway [101]. In addition, in hepatocellular carcinoma, PH domain and leucine-rich repeat protein phosphatase 1 (PHLPP1), an AKT phosphatase, was ubiquitinated in a K48-linked manner by TRIM11, leading to its degradation and an increase in phospho-AKT levels, thus activating the PI3K/AKT signaling pathway [102]. In gastric cancer, overexpression of TRIM14 enhanced aggressiveness by promoting EMT and metastasis in MKN45 and SGC7901 cells. Its overexpression also increased lung metastases in vivo in BALB/c nude mice [103]. These effects are mediated by activation of AKT signaling, as there was a positive correlation between TRIM14 and phospho-AKT, and AKT inhibition can reverse TRIM14-induced cancer cell invasion and migration [103]. Furthermore, TRIM14 expression was regulated by miR-195-5p, and its overexpression was linked to lower miR-195-5p levels in gastric cancer tissues [103]. In addition, elevated TRIM14 levels can localize PTEN to the cytoplasm and induce polyubiquitination to degrade it in colorectal cancer, thereby triggering AKT signaling. This effect was confirmed in vitro using HT-29 and SW620 colorectal cancer cells, where TRIM14 knockdown increased PTEN levels and reduced AKT phosphorylation, and results of xenograft models in BALB/c nude mice showed reduced tumor growth following TRIM14 silencing. In sum, TRIM14 promotes the PI3K/AKT pathway by reducing PTEN, enhancing cancer cell invasiveness [104].

In vitro, using SGC-7901 and MKN-1 gastric cancer cells, upregulation of TRIM24 protein levels induced chemoresistance to 5-Fluorouracil (5-FU) and led to an increase in phospho-AKT and cyclin D1, an effect that was counteracted by an AKT inhibitor, suggesting that TRIM24 activated the AKT pathway to induce this chemoresistance [105]. As mentioned above, TRIM24 can be downregulated by miR-511, thus inhibiting the PI3K/AKT pathway [73]. In glioma, TRIM24 activated *PIK3CA* gene expression by binding to its promoter via the PHD–bromodomain, thus triggering the PI3K/AKT pathway [106]. Similarly, TRIM24 can increase both *PIK3CA* and *EGFR* levels in human prostate cancer cell lines (LNCaP, PC-3, and C4-2), thereby facilitating the activation of the PI3K/AKT signaling pathway. Additionally, Linc00963 promotes TRIM24 expression in castration-resistant prostate cancer cells by suppressing miR-655 [107]. Taken together, TRIM24 enhances the PI3K/AKT pathway by acting at multiple levels, from gene transcription to protein stability, promoting cancer cell survival and treatment resistance. TRIM27 promoted AKT/mTOR signaling by mediating the small ubiquitin-like modifier conjugation (SUMOylation), a post-translational modification, of Tuftelin (TUFT1) at lysine 79, as evidenced by in vitro experiments in AGS/HGC27 cells and in vivo studies using subcutaneous xenograft models. This post-translational modification was critical for pathway activation and the oncogenic function of TUFT1 [108]. The knockdown of TRIM32 in gastric cancer cells, including NCI-N87, MKN74, and MKN45, resulted in increased apoptosis and decreased proliferation and AKT phosphorylation, which can be further enhanced by AKT inhibitors. Moreover, silencing TRIM32 significantly suppressed tumor growth in a subcutaneous xenograft model using nude mice. These findings suggest that TRIM32 may facilitate gastric cancer progression by promoting AKT phosphorylation, thus activating the PI3K/AKT pathway [109].

Conclusively, TRIM proteins play a critical role in the PI3K/AKT pathway through various mechanisms, such as affecting the phosphorylation state and activity of AKT and downstream targets to modulate the metabolism, proliferation, and survival of gastric cancer development.

### 3.3. TGF-β/Smad Signaling Pathway

#### 3.3.1. Overall Role and Mechanism of TGF-β/Smad Signaling Pathway

Transforming Growth Factor-β (TGF-β) significantly affects gastric cancer, with increased levels observed in patients, promoting lymph node metastasis, along with mutation in *TGFB1* and *TGFB2* [110,111,112]. In the premalignant stage, TGF-β usually suppresses tumor progression by maintaining homeostasis and regulating tumor cell proliferation, differentiation, and cellular microenvironment. While in the malignant stage, pathological TGF-β signal transduction shifts towards promoting invasion, metastasis, tumor cell growth, and immune evasion, specifically by facilitating Smad-dependent EMT [113,114,115]. In the Smad-dependent TGF-β pathway, type I and type II receptors bind together in response to TGF-β, phosphorylating Smad2 and Smad3 (R-Smads). Then, they come together with Smad4 (Co-Smad), forming a complex, and subsequently enter into the nucleus and modulate target genes [113,116,117].

#### 3.3.2. TRIM Proteins Act as Activators of the TGF-β Signaling Pathway

Several TRIM proteins have been found to activate this pathway. TRIM25 was overexpressed in gastric cancer tissues and cell lines (MGC-803 and AGS). In vitro, its silencing inhibited cancer cell migration and invasion and reduced phospho-Smad2 and phospho-Smad4 levels, while its ectopic expression promoted gastric cancer progression and counteracted TGF-β inhibitors. This suggests that TRIM25 facilitates the invasion and migration of cancer cells by triggering the TGF-β pathway [118].

#### 3.3.3. TRIM Proteins Act as Inhibitors of the TGF-β/Smad Pathway

Some TRIM proteins can inhibit this pathway. TRIM22 had a low expression level in gastric cancer tissues and cells. In vitro, its overexpression decreased phospho-Smad2 and phospho-Smad3 in 746T and AGS cells. Moreover, the anti-growth effect of TRIM22, which was observed in subcutaneous xenograft experiments, can be reversed by the upregulation of Smad2, indicating that TRIM22 suppressed gastric cancer progression by blocking the TGF-β/Smad pathway [119]. TRIM33 is typically overexpressed in gastric cancer. However, its downregulation led to the promotion of proliferation, colony formation, EMT, and migration of gastric cancer cells (BGC-823 and SGC-7901), along with increased TGF-β signaling pathway-related proteins, including TGF-β, phospho-Smad2, Smad2, Smad3, and Smad4. Results of subcutaneous xenograft models demonstrated that TRIM33 knockdown promoted tumor growth, suggesting that TRIM33 inhibited TGF-β signaling to exert its tumor-suppressive role [120]. Meanwhile, upon binding to Smad2/3, TRIM33 competes with Smad4 in pancreatic cancer, leading to a different effect from the classical complex. TRIM33 knockdown enhanced Smad4-mediated transcription in PANC-1 cells. In vivo studies in mouse models with orthotopic pancreatic cancer demonstrated that TRIM33 suppressed tumor progression by modulating TGF-β signaling. TRIM33 also mediates monoubiquitination of Smad4 at lysine 519, thus inhibiting the TGF-β/Smad signaling pathway in HEK293T and HaCaT cells [121,122]. These findings collectively indicate that TRIM33 suppresses tumor progression in various cancers by intervening at multiple levels of the TGF-β/Smad pathway. Protein phosphatase Mg^2+^/Mn^2+^-dependent 1A (PPM1A) can promote Smad2/3 dephosphorylation, thereby suppressing TGF-β/Smad signaling [123,124]. In gastric cancer, TRIM65 expression was elevated in both clinical tumor tissues and cell lines. TRIM65 was shown to promote the ubiquitin-mediated degradation of PPM1A in AGS and SNU-1 cells. The suppression of PPM1A counteracted the inhibitory effects of TRIM65 knockdown on cancer progression, suggesting that TRIM65 facilitates gastric cancer progression by degrading PPM1A. Thus, TRIM65 activates TGF-β/Smad signaling by alleviating its inhibition, thereby promoting cancer cell growth and progression [125].

In conclusion, through the regulation of key molecules in the TGF-β/Smad signaling pathway, members of the TRIM family proteins can promote or suppress metastasis, invasion, and proliferation of gastric cancer cells, indicating their potential as therapeutic targets.

### 3.4. Other Signaling Pathways

Beyond the three main signaling pathways discussed, TRIM proteins also influence gastric cancer progression through other pathways, which may also be crucial for tumor development.

#### 3.4.1. P53 Signaling Pathway

The p53 pathway is critical for apoptosis, DNA repair, and cell cycle control [126]. It regulates cellular responses to damage by activating key genes like *p21* and *Reprimo*, which arrest checkpoint regulation at G1/S and G2/M during cell cycle progression, thereby facilitating repair and preventing malignant cell proliferation [127,128]. Therefore, p53 is crucial in preventing tumor formation.

In gastric cancer, TRIM29 silencing led to elevated p53 and p21, cell cycle arrest, and cell proliferation inhibition in SGC-7901 and MGC80-3 cells. Moreover, results of subcutaneous xenograft models further confirmed that TRIM29 silencing significantly reduced tumor growth. Mechanistically, TRIM29 exhibits its oncogenic role by suppressing p53 transcriptional activity, thus inhibiting the p53 pathway [75]. In gastric cancer, high expression of TRIM36 can increase the OS rate in gastric cancer patients receiving radiotherapy. This finding was based on a clinical study using patient data from The Cancer Genome Atlas (TCGA), which demonstrated that TRIM36 inhibited the p53 signaling pathway. Stratified analyses of radiotherapy groups further confirmed that patients with high TRIM36 expression showed increased radiosensitivity [129]. This indicates that TRIM36 may play a dual role, acting as a radiosensitizer while inhibiting the p53 pathway, potentially improving outcomes for specific patient groups. Additionally, in gastric cancer, TRIM59 was highly expressed in tumor tissues and gastric cancer cell lines (AGS and MKN45), which promoted the growth of tumors in vivo in BALB/c nude mice bearing subcutaneous xenografts and the proliferation of cancer cells in vitro [130]. Mechanistically, TRIM59 mediates p53 ubiquitination and increases degradation, leading to inactivation of the p53 pathway [130]. Overall, these findings reveal that TRIM proteins play a complex role in gastric cancer by regulating the p53 pathway.

#### 3.4.2. JAK-STAT Signaling Pathway

The Janus Kinase/Signal Transducer and Activator of Transcription (JAK-STAT) pathway impacts crucial physiological processes like immune response, proliferation, and metabolism [131]. Upon binding of ligands like interleukins (ILs) and interferons (IFNs) to specific cell surface receptors, the receptor-associated JAK kinases are activated, recruiting and phosphorylating STATs [132]. Following phosphorylation, these STATs translocate to the nucleus as dimers to control gene expression [132,133].

In gastric cancer, TRIM19 expression was decreased or absent in clinical tumor tissues, which correlated with higher T lymphocyte infiltration [134]. Mechanistically, knockdown of TRIM19 in SNU-638 cells activated the IFN-γ-mediated JAK/STAT pathway by enhancing the attachment of STAT to the promoter of interferon-gamma-inducible protein 10 (IP-10), thereby promoting the expression of IP-10, a cytokine recruiting natural killer (NK) cells and T cells [134,135]. These findings suggest that TRIM19 may act as a tumor suppressor by promoting immune cell infiltration through the JAK/STAT pathway. Compared with nearby normal tissues, TRIM21 expression was reduced in gastric cancer, which was mediated by STAT1. In vitro, the interaction between STAT1 and the TRIM21 promoter was confirmed in 293FT cells, and in vivo, TRIM21 overexpression in MKN45 cells suppressed tumor growth in nude mice, aligning with its tumor-suppressive role in gastric cancer [136]. In turn, TRIM21 functioned as an E3 ligase targeting STAT1 for ubiquitin-mediated degradation, thereby inhibiting the JAK/STAT pathway, which suppressed cell migration and proliferation while promoting apoptosis. These findings, derived from in vitro experiments on human gastric cancer cell lines AGS and MKN28, indicated a potential regulatory loop between TRIM21 and STAT1 [137]. Upregulation of TRIM28 was found in gastric cancer cells and contributed to reduced OS rate. As validated through evidence from patient tissues and in vitro experiments using AGS and MKN45 cell lines, TRIM28 activated the transcription of STAT3, thus triggering the JAK/STAT pathway and promoting gastric cancer cell metastasis [138]. Overall, these findings reveal that TRIM proteins regulate the JAK/STAT pathway in gastric cancer through mechanisms such as promoting immune cell infiltration, modulating cytokine expression, and activating or inhibiting transcriptional activity.

#### 3.4.3. NF-κB Signaling Pathway

The nuclear factor kappa-light-chain-enhancer of activated B cells (NF-κB) signaling pathway is integral to numerous physiological processes, including inflammation, cellular growth, and immune response [139]. Within the canonical NF-κB pathway, tumor necrosis factor receptor 1 (TNFR1) receives and delivers signals that induce the binding of tumor necrosis factor receptor type 1-associated death domain protein (TRADD). TNF receptor-associated factor 2 (TRAF2) and Fas-associated protein with death domain (FADD) are recruited, which then bind with receptor-interacting proteins (RIPs) [139]. A critical step involves phosphorylation of the IκB kinase (IKK) complex, including a regulatory subunit, NF-kappaB essential modulator (NEMO), and two catalytic subunits, IKKα and IKKβ, which promotes the phosphorylation, ubiquitination, and degradation of inhibitor of κB (IκB) [139,140,141]. In resting status, NF-κB dimers (usually p50:p65) are attached to inhibitory IκB proteins, confining NF-κB complexes within the cytoplasm [139]. Upon activation, NF-κB dimers are released due to IκB degradation, allowing them to get into the nucleus and trigger target gene transcription [142].

There was higher TRIM37 expression in MKN-45 and HGC-27 cells. LncRNA *ASB16-AS1* facilitated the phosphorylation and expression of TRIM37 by binding with miR-3918/miR-4676-3p, thus activating the NF-κB signaling pathway, which in turn promoted cell stemness, proliferation, and chemoresistance to cisplatin in HGC-27 and MKN-45 cells and xenograft mouse models [143]. There was an increased expression level of TRIM40 in gastrointestinal carcinoma. Functional studies demonstrated that TRIM40 overexpression facilitated neddylation of IKKγ, a post-translational modification attaching neural precursor cell-expressed, developmentally downregulated 8 (NEDD8) to proteins and impacting their stability and function, and stabilized IκBα in HEK293T, HeLa, and SW480 cells. Conversely, inhibition of TRIM40 had the opposite effect to promote cell proliferation. This suggests that TRIM40 functions as a tumor suppressor by negatively regulating the NF-κB pathway through the neddylation of IKKγ [144]. TRIM47 was overexpressed in clinical gastric cancer tissues, which is linked to poor differentiation and advanced TNM stages (III/IV) by bioinformatics analysis. The promotive effect of TRIM47 on gastric cancer can be mediated by the NF-κB signaling pathway in vitro in experiments using AGS gastric cancer cell lines [145]. Mechanistically, TRIM47 can interact with IκBα protein, whose upregulation reduces IκBα expression via ubiquitination and subsequent degradation, promoting p65 translocation from the cytoplasm to the nucleus, activating the NF-κB pathway. This mechanism was supported by both in vitro experiments using AGS and MKN45 gastric cancer cell lines and in vivo studies involving tail vein injection of MKN45 cells into BALB/c nude mice [146]. TRIM59 levels were decreased in human gastric cancer tissues, which was associated with a favorable prognosis in patients. As supported by analyses of gastric cancer patient tissues and in vitro experiments using human gastric cancer cell lines BGC823 and SGC7901, TRIM59 promoted the degradation of TRAF6, a member of the TRAF family, via ubiquitination, thus inhibiting the NF-κB signaling pathway [147]. Collectively, these findings reveal that TRIM proteins regulate the NF-κB pathway in gastric cancer through mechanisms such as modulating protein ubiquitination, stabilizing key pathway components, and interacting with non-coding RNAs.

#### 3.4.4. Others

The Hippo pathway is important for modulating tissue growth and regeneration [148]. The kinase cascade at the center of this pathway is made up of large tumor suppressor 1 (LATS1) and 2 (LATS2), as well as mammalian STE20-like protein kinase 1 (MST1) and 2 (MST2), which mediates the phosphorylation of Yes-associated protein (YAP) and transcriptional co-activator with PDZ-binding motif (TAZ), blocking their entry into the nucleus and thus inhibiting cell proliferation [148]. With an overexpressed level in human gastric cancer tissues, TRIM27 facilitated the tumor progression by activating the Hippo-BIRC5 (baculoviral inhibitor of apoptosis repeat containing 5) axis [149]. TRIM27 knockdown in MGC-803 and HGC-27 cells enhanced the protein levels of LATS2 and phospho-YAP1 and reduced the expression of BIRC5 and its product, Survivin. However, in YAP1-deficient cells, TRIM27 suppression did not affect BIRC5 or Survivin expression, indicating YAP1 dependency [149]. Mechanistically, TRIM27 downregulated LATS2, potentially through LATS2 ubiquitination and subsequent degradation. This led to reduced YAP1 phosphorylation, facilitating its nuclear translocation and activating its downstream target, BIRC5 [149].

Functioning as an essential energy sensor, Adenosine Monophosphate-Activated Protein Kinase (AMPK) controls the balance of energy within cells by monitoring ATP and AMP levels, which is crucial in metabolic processes and systemic energy equilibrium [150]. As an upstream regulator of the mTOR pathway, AMPK activation reduces mTOR signaling, a mechanism vital for energy conservation and cellular stress response [150]. In gastric cancer cells resistant to 5-FU and OXA, TRIM14 was found to be overexpressed, whose oncogenic effect was mediated by triggering the AMPK/mTOR pathway [150]. Upregulation of TRIM14 had a promotive effect on autophagy and proliferation and an inhibitory effect on apoptosis in SGC7901/5-FU cells, while TRIM14 knockdown had the reverse effect both in vitro and in vivo in a subcutaneous xenograft model of 5-FU-resistant gastric cancer in BALB/c nude mice, which can be reversed by MK3903, an agonist of the AMPK/mTOR pathway [150].

## 4. Role of TRIM Proteins in Gastric Cancer Development Independent of Signaling Pathways

It has been shown that numerous TRIM family proteins exert influence over the onset and progression of gastric cancer via non-signaling mechanisms. These proteins can affect essential cellular activities, including apoptosis, differentiation, and the cell cycle. This is achieved through multiple approaches, such as alteration of gene transcription, participation in protein degradation, regulation of protein stability, modulation of cellular functions through protein–protein interactions, etc. (Table 1).

### 4.1. Oncogenic Roles of TRIM Proteins in Gastric Cancer

TRIM family members, including TRIM15, TRIM16, TRIM23, TRIM29, TRIM31, TRIM32, TRIM44, TRIM47, and TRIM55, are significantly upregulated in human gastric cancer cell tissues and are highly associated with poor prognosis. Through in vitro experiments, it has been demonstrated that these proteins promote cancer cell invasion, migration, and proliferation, thereby accelerating tumor progression. TRIM16, significantly upregulated in tissues from stage IV gastric cancer patients, is directly regulated by the lncRNA SDMGC. TRIM16 knockdown resulted in a marked reduction in migration and invasion in AGS and SGC7901 cells, whereas its overexpression enhanced these malignant properties. Additionally, several TRIM proteins, including TRIM15, TRIM47, and TRIM55, have been implicated in EMT. These proteins facilitate EMT by modulating key molecular markers such as E-cadherin, N-cadherin, and Vimentin, contributing to increased cancer cell invasiveness and metastatic potential [74,75,76,145,151,152,153,154,155,156,157,158,159].

Meanwhile, some TRIM proteins influence gastric cancer progression through the regulation of substrates or transcription factors. Gastric cancer tissues had a notable overexpression of TRIM17, as shown by tissue microarray (TMA) analysis of clinical patient samples. Overexpression of TRIM17 in AGS and HGC-27 cells enhanced the proliferation and survival of tumor cells, whereas its suppression resulted in increased apoptosis. Furthermore, knockdown of TRIM17 in AGS cells suppressed tumor growth in a xenograft mouse model. Mechanistically, TRIM17 ubiquitinated and degraded BAX, a pro-apoptotic protein, resulting in decreased BAX-dependent apoptosis [160]. In gastric cancer specimens, there was a notable increase in TRIM28 expression in clinical tumor samples, which was associated with poor prognosis, larger tumor size, and increased peritoneal carcinomatosis [161,162,163]. Reducing TRIM28 levels in MKN45 and MKN28 cells suppressed cell proliferation and increased sensitivity to anoikis [163]. Furthermore, TRIM28 has been found to facilitate gastric cancer cell proliferation by the serum response factor/Indoleamine 2,3-dioxygenase (SRF/IDO1) axis, whereby it regulates IDO1 expression by modulating SRF levels. TRIM28 knockdown notably inhibited gastric cancer progression through the suppression of IDO1 both in vitro using MGC803 and 746T cells and in vivo via a subcutaneous xenograft model in BALB/c nude mice injected with MGC803 cells, while overexpression of IDO1 or SRF can counteract the TRIM28 knockdown-induced reduction in proliferation [161]. As an oncogenic protein in gastric cancer, TRIM37 was expressed at higher levels in human gastric cancer tissues than in peritumoral tissues [164,165]. Analyses of clinical samples confirmed that overexpression of TRIM37 led to poor prognosis and increased incidence of lymphatic recurrence. In vitro experiments using MKN45 and AGS cells demonstrated that elevated TRIM37 correlated with aggressive characteristics, including invasion and advanced stage in gastric cancer, while TRIM37 knockdown had the opposite effects [164,165]. In vivo, a subcutaneous xenograft model in BALB/c nude mice revealed that TRIM37 silencing reduced tumor growth. Mechanistically, TRIM37 can facilitate the invasion and metastasis of gastric cancer cells by increasing the expression of Smad-interacting protein 1 (SIP1), a transcription factor that regulates EMT. Conversely, silencing of TRIM37 had been proved to have the opposite effects, suggesting its potential significance in gastric cancer progression via SIP1 [164,166]. A significantly upregulated level of TRIM54 was observed in human gastric cancer tissues, which was closely associated with advanced tumor stages, increased metastasis, and poorer overall survival. Overexpression of TRIM54 can facilitate cancer progression by promoting cell proliferation, invasion, and migration in AGS and HGC27 cells, and its knockdown suppressed gastric cancer progression in vivo in a xenograft model [167]. Functional studies revealed that TRIM54 acted as a post-translational mediator for filamin C (FLNC), an actin-binding protein, promoting its K63-linked ubiquitination and subsequent degradation. This interaction suggested a regulatory mechanism where TRIM54 overexpression exerted a positive influence on gastric cancer aggressiveness [167].

Collectively, these TRIM proteins demonstrate how different mechanisms, such as regulation of apoptosis, transcription factors, and cytoskeletal proteins, contribute to increased tumor aggressiveness. Future research focusing on their upstream and downstream interactions and effects on the immune microenvironment or treatment sensitivity may help identify more effective intervention strategies.

### 4.2. Suppressive Roles of TRIM Proteins in Gastric Cancer

Interestingly, TRIM15 and TRIM16 not only show higher expression in gastric cancer tissues, promoting cancer progression, but also exhibit lower expression in some studies, where they inhibited cancer progression [168,169]. There was a notable decrease in TRIM19 expression levels in clinical gastric cancer tissues, associated with increased lymphatic invasion and advanced cancer stages, which ultimately correlated with poorer patient outcomes [170]. Although TRIM31 had been identified as being overexpressed in gastric adenocarcinoma, as confirmed in patient-derived tissue samples, in vitro experiments using HCT116 and AsPC-1 cells revealed that it suppressed cell proliferation and colony formation; conversely, its knockdown had the opposite effects, suggesting its potential tumor-suppressor properties [171]. The level of TRIM31 was influenced by multiple regulatory factors, such as proteasome-mediated degradation and inducible transcription, which may explain its contradictory roles in gastric cancer [172].

Moreover, many TRIM proteins also influence gastric cancer progression by regulating the activity of substrates or transcription factors. A reduced level of TRIM7 was observed in human gastric cancer tissues, and its overexpression was associated with prolonged patient survival rates [173]. Mechanistically, TRIM7 induced ferroptosis and suppressed gastric cancer progression by targeting Solute Carrier Family 7 Member 11 (SLC7A11), facilitating Lys48-linked polyubiquitination through its B30.2/SPRY domain, thus inhibiting glutathione peroxidase 4 (GPX4) level, which is crucial for cellular antioxidant defenses. These findings were validated through in vitro experiments using AGS and MKN45 cells and in vivo xenograft models established in immunodeficient nude mice [173]. TRIM21 expression was markedly downregulated in tumor tissues of gastric cancer patients [136]. Clinical samples from the study showed that reduced TRIM21 expression correlated with advanced tumor stages, higher recurrence rates, and poor survival outcomes [174]. As a tumor suppressor, overexpression of TRIM21 suppressed cell migration and proliferation, facilitated apoptosis in AGS and MKN45 cells, and limited tumor growth in nude mouse xenograft models [136]. Meanwhile, TRIM21 also suppressed the expression of enhancer of zeste homolog 1 (EZH1), a protein playing a role in maintaining chromatin compaction, which contributes to the chemosensitivity of tumor cells in SGC7901 and BGC823 cells, where TRIM21 overexpression reduced EZH1 stability and expression levels, enhancing tumor cell chemosensitivity. This, in turn, had been found to increase the susceptibility of gastric cancer cells to apatinib treatment in vivo using male nude mice subcutaneously injected with SGC7901 cells, which is capable of inhibiting gastric cancer development, suggesting the potential therapeutic value of TRIM21 in gastric cancer [174,175,176]. Collectively, TRIM21 acts as a tumor suppressor in gastric cancer by inhibiting tumor growth, migration, and proliferation while enhancing apoptosis and chemosensitivity through the downregulation of EZH1. Furthermore, TRIM21 was related to the tumor microenvironment (TME), which can directly impact B-cell growth and development and enhance membrane antibody trafficking [177]. In addition, TRIM21 was associated with the presence of different immune cells, including NK cells, T cells, and dendritic cells in gastric cancer, as demonstrated by bioinformatics analysis [136]. TRIM25 was under-expressed in human gastric cancer tissues, whose downregulation was associated with poor prognosis of patients. TRIM25 inhibits MMP2 expression by mediating the ubiquitination at K610 and degradation of SP1, the transcription factor of MMP2, thereby suppressing angiogenesis in gastric cancer. Overexpression of TRIM25 in human gastric cancer cell lines (BGC823, SGC7901, and MGC803) reduced angiogenesis and MMP2 expression. TRIM25 was found to be stabilized by JP3 through phosphorylation at Ser12. The results of subcutaneous xenograft models further confirmed TRIM25’s anti-angiogenic role, as evidenced by decreased microvessel density and reduced tumor growth [178].

Notably, another research team found that TRIM29 was upregulated in human gastric cancer tissues. Reduced expression of TRIM29 in gastric cancer samples was associated with advanced tumor stages, lower CD8^+^ immune cell infiltration, and poorer survival outcomes in clinical sample analysis [179]. Programmed death ligand 1 (PD-L1), an oncogenic ligand, was increased in carcinogenesis, with mRNA stability maintained by insulin-like growth factor 2 mRNA-binding protein 1 (IGF2BP1) [179,180]. TRIM29 promoted the degradation of IGF2BP1 by mediating its K48-type ubiquitination at the Lys440 and Lys450 sites, thereby decreasing the PD-L1 level and acting as a tumor suppressor to promote antitumor T-cell immunity. This regulatory role was demonstrated in vitro using gastric cancer cell lines (AGS and MKN45) and validated in vivo with xenograft tumor models in BALB/c nude mice [179]. This is in stark contrast to previous studies suggesting TRIM29 as an oncogene. It highlights the necessity for further studies to reveal the specific role of TRIM29 in gastric cancer development.

The clinical analysis of gastric cancer samples showed that high TRIM44 expression was associated with advanced TNM stages, reduced T-cell infiltration, and poor overall survival. It promoted T-cell-mediated antitumor immunity through the regulation of matrix alterations [181]. Specifically, TRIM44 mediated the ubiquitination and subsequent degradation of Lysyl Oxidase Like 2 (LOXL2), thus impacting extracellular matrix remodeling and influencing tumor immunity in gastric cancer. This has been confirmed through in vitro studies in AGS and SGC-7901 cells and in vivo experiments using subcutaneous tumor models in immunocompetent 615 mice and Rag1 KO mice. Thus, TRIM44 regulates ECM components, altering the immune microenvironment and linking matrix remodeling to immune cell infiltration [181,182]. TRIM50 was under-expressed in gastric cancer tissues, which was associated with larger tumor size, advanced TNM stages, and poorer survival outcomes in clinical analysis. Its tumor-suppressive function of inhibiting tumor growth and migration was validated in vitro in MKN45 and SNU-668 cells and in vivo experiments involving subcutaneous xenografts and dissemination models in BALB/c nude mice. Mechanistically, TRIM50 inhibited tumor development by targeting junction plakoglobin (JUP), a transcription factor, for degradation mediated by K63-linked ubiquitination at the K57 site. This was crucial for allowing JUP to translocate into the nucleus, thus effectively restraining the MYC pathway [183]. In addition, TRIM50 suppressed glycolysis and tumor progression by mediating ubiquitination and degradation of Phosphoglycerate Kinase 1 (PGK1), a key enzyme in the glycolytic pathway [184]. This inhibition not only suppressed gastric cancer cell proliferation directly in HGC-27 cells and in vivo through a subcutaneous xenograft mouse model but also indirectly mitigated cell invasion and migration through its impact on tumor-associated macrophage M2 polarization in vitro [184]. Furthermore, the downregulation of TRIM50 in gastric cancer cells was mediated by the METTL3/YTHDF2 axis via m6A methylation. Taken together, TRIM55 suppresses tumor growth, migration, and glycolysis by mediating K63-linked ubiquitination of JUP and PGK1, inhibiting the MYC pathway and glycolytic activity [184]. TRIM69 expression was notably suppressed in gastric cancer cells resistant to anoikis, which was correlated with advanced tumor stages, enhanced metastasis, and poorer prognosis as shown by the clinical analysis [185]. Using AR AGS, MKN28, MKN45, and HGC27 cells in vitro and lung metastasis assays in BALB/c athymic nude mice in vivo, experiments have revealed that overexpression of TRIM69 led to decreased anoikis resistance and reduced metastatic potential [185]. This effect was mediated by polyubiquitination at the K48 site and degradation of protein kinase C delta (PRKCD), thus inhibiting the expression of brain-derived neurotrophic factor (BDNF) [185].

In summary, TRIM proteins suppress gastric cancer progression through mechanisms such as regulation of metabolism, cell survival, and ECM remodeling. Their diverse functions provide new insights into non-signaling mechanisms of tumor suppression.

**Table 1:** Role, effect, and mechanism of TRIM proteins in gastric cancer.

Name	Role	Effect	Mechanism	Reference
TRIM3	Suppressive	↓ Growth	Inhibits *BCL2*, *β-catenin*, and *cyclin D* gene expression	[83]
		↓ Metastasis	Regulates the stem cell factors and EMT regulators	[85]
TRIM7	Suppressive	↑ Ferroptosis	Suppresses the SLC7A11/GPX4 axis	[173]
TRIM11	Oncogenic	↑ Migration ↑ Invasion	Decreases Axin1 stability to activate the Wnt/β-catenin pathway	[69,70]
		↑ Proliferation	Contributes to activation of the AKT signaling pathway	[101]
TRIM14	Oncogenic	↑ Autophagy	Stabilizes Dvl2 to activate the Wnt/β-catenin signaling pathway	[71]
		↑ Chemoresistance	Activates the AMPK/mTOR pathway	[150]
		↑ Metastasis	Activates the AKT signaling pathway	[103]
TRIM15	Oncogenic	↑ Invasion ↑ Migration	-	[151]
	Suppressive	↓ Invasion	-	[168]
TRIM16	Suppressive	↓ Proliferation ↓ Invasion ↓ Migration	Low expression leads to *β-catenin*, *cyclin D*, and *BCL2* gene accumulation	[84,169]
	Oncogenic	↑ Invasion ↑ Migration	-	[152]
TRIM17	Oncogenic	↑ Apoptosis	Promotes BAX degradation	[160]
TRIM19	Suppressive	↓ Lymphocyte infiltration	Controls IP-10 expression through modifying the IFN-γ-mediated JAK/STAT pathway	[134,170]
TRIM21	Suppressive	↓ Proliferation ↓ Migration ↑ Apoptosis	Promotes STAT1 degradation	[136,137]
		↑ Chemosensitivity	Suppresses the expression of EZH1 protein	[174]
TRIM22	Suppressive	↓ Proliferation	Inhibits the TGF-β/Smad pathway	[119]
TRIM23	Oncogenic	-	-	[153]
TRIM24	Oncogenic	↑ Proliferation ↑ Chemoresistance	Activates the Wnt/β-catenin pathway and the AKT pathway	[72,73,105]
TRIM25	Oncogenic	↑ Migration ↑ Invasion	Activates the TGF-β pathway	[118]
	Suppressive	↓ Angiogenesis	Promotes the degradation of SP1	[178]
TRIM27	Oncogenic	↑ Proliferation	Induces the degradation of LATS2 to activate the Hippo-BIRC5 axis	[149]
			Mediates SUMOylation of TUFT1 to activate the AKT/mTOR pathway	[108]
TRIM28	Suppressive	↓ Stem-like property of GC cells	Knockdown activates the Wnt/β-catenin pathway	[86]
	Oncogenic	↑ Metastasis	Activates the JAK/STAT pathway by promoting the transcription of STAT3	[138]
		↑ Proliferation	Regulates SRF/IDO1 axis	[161]
		↑ Metastasis	-	[162]
		↑ Peritoneal dissemination	-	[163]
TRIM29	Oncogenic	↑ Proliferation ↑ Invasion ↑ Migration	Inhibits the p53 signaling pathway	[75]
		↓ Apoptosis	β-catenin/CyclinD/Bcl2 pathway	[74]
	Suppressive	↑Antitumor immunity	TRIM29/IGF2BP1/PD-L1 axis	[179]
TRIM31	Suppressive	↓ Proliferation	-	[171,172]
	Oncogenic	↑ Invasion ↑ Proliferation	Activates the Wnt/β-catenin pathway	[65]
TRIM32	Oncogenic	↑ Invasion	Activates the Wnt/β-catenin pathway	[78]
		↑ Glycolysis	Participates in AKT-GLUTI/HKII signaling pathway	[109]
		↑ Proliferation	-	[157]
TRIM33	Suppressive	↓ Survival ↓ EMT	Downregulation activates the TGF-β signaling pathway	[120]
TRIM36	-	↑ Radiosensitivity	High expression may inhibit the p53 pathway	[129]
TRIM37	Oncogenic	↑ Invasion	ASB16-AS1 activates the NF-κB pathway through TRIM37	[143,165]
		↑ Chemoresistance	Activates SIP1-induced EMT	[164]
TRIM40	Suppressive	↓ Tumor growth	Inhibits the NF-κB pathway	[144]
TRIM44	Suppressive	↑ Tumor immunity	Decreases LOXL-2 stability	[181]
	Oncogenic	↑ Tumorigenesis	Promotes β-catenin signaling	[80,81]
		↑ Gastric cancer stem cell survival	-	[158]
TRIM47	Oncogenic	↑ Migration ↑ Invasion	Activates the NF-κB pathway	[145,146]
TRIM50	Suppressive	↓ Proliferation	Suppresses the MYC pathway by promoting degradation of JUP	[183]
		↓ Metastasis	Blocks the Wnt/β-catenin pathway	[87]
		↓ Glycolysis	Mediates the ubiquitination of PGK1	[184]
TRIM52	Oncogenic	↑ Proliferation ↑ Migration ↑ Invasion	Activates the Wnt/β-catenin pathway	[82]
TRIM54	Oncogenic	↑ Growth ↑ Metastasis	Mediates the degradation of filamin C	[167]
TRIM55	Oncogenic	↑ Metastasis	-	[159]
TRIM58	Suppressive	↓ Tumor growth	Inactivates the Wnt/β-catenin pathway	[88]
TRIM59	Oncogenic	↑ Tumor growth	Inhibits the p53 pathway	[130]
		↑ Proliferation ↑ Migration ↑ Invasion	Promotes TRAF6 degradation via ubiquitination	[147]
TRIM65	Oncogenic	↑ Proliferation ↑ Invasion	Knockdown suppressing the UPS-mediated PPM1A degradation	[125]
TRIM69	Suppressive	↓ Anoikis resistance ↓ Metastasis	Mediates the ubiquitination and degradation of PRKCD to inhibit BDNF production	[185]

↑: increased; ↓: decreased.

## 5. Clinical Significance and Future Perspectives of TRIM Proteins in GC

With E3 ligase activity, TRIM family proteins have been discovered to contribute to the progression of many cancers, including breast cancer, hepatocellular carcinoma, and lung cancer [186,187,188]. Recently, a developing number of studies have highlighted the critical functions that TRIM proteins play in the initiation, progression, and treatment of gastric cancer.

Undoubtedly, the expression of TRIM proteins in gastric cancer has notable clinical significance (Table 2). Higher or lower expression of various TRIM proteins is not only relative to the OS of patients and other prognostic factors but also linked to many clinicopathological characteristics. Upregulation of specific TRIM proteins, including TRIM11, TRIM14, TRIM23, TRIM24, TRIM29, TRIM31, and TRIM37, and downregulation of TRIM19 are strongly linked to metastasis, invasion, and TNM staging in gastric cancer patients. All of these are associated with poor prognosis, including shorter survival periods, lower response to drug treatment, and higher risk of metastasis and recurrence. Therefore, TRIM proteins can be prognostic factors for gastric cancer, helping to predict disease progression and guide adjustments in treatment strategies.

Gastric cancer is characterized by late diagnosis and poor prognosis, which limits the effectiveness of conventional treatments. Thus, identifying effective therapeutic targets is currently a focus of research. Specifically, as shown in Table 1, proteins like TRIM11, TRIM14, TRIM24, TRIM28, TRIM32, TRIM37, and TRIM44 have been identified as positive modulators of EMT, migration, invasion, and proliferation in gastric cancer. TRIM11 and TRIM47 were found to promote gastric cancer cell invasion and migration; TRIM14, TRIM24, and TRIM37 contributed to chemoresistance; TRIM27 and TRIM59 effectively promoted cell proliferation. Furthermore, increased TRIM32 level was found to correlate with enhanced glycolysis, a common metabolic pathway in cancer cells. In addition, TRIM3, TRIM21, TRIM31, and TRIM50 inhibited cell proliferation and metastasis, contributing to the suppression of gastric cancer development (Table 1). For instance, in gastric cancer, overexpressed TRIM3 and TRIM50 were found to suppress tumor growth and metastasis; similarly, elevated levels of TRIM21 enhanced the sensitivity to apatinib in gastric cancer. Notably, TRIM29 appears to have a dual regulatory effect with different experimental results reported. In addition to regulating cell behaviors, TRIM proteins also modulate the tumor microenvironment. For instance, TRIM29 enhances antitumor immunity by promoting the ubiquitination of IGF2BP1, leading to the downregulation of PD-L1 expression. This mechanism fosters the activity of CD8^+^ T cells, thus improving immune-mediated tumor suppression [179]. On the other hand, TRIM44 promoted antitumor immunity by mediating the degradation of LOXL2. This process facilitates T-cell infiltration and immune response, ultimately suppressing tumor progression [181].

With the advances in research on TRIM proteins, targeting these proteins could regulate TRIM protein expression in gastric cancer, thereby inhibiting cancer progression and improving chemotherapy sensitivity. Several studies have revealed that specific TRIM proteins can be regulated by targeted compounds in various cancers. For example, arsenic trioxide suppresses TRIM19 in glioblastoma, Withaferin A enhances TRIM16 activity in melanoma, and eugenol inhibits TRIM59 in non-small cell lung cancer [189,190,191]. These findings underscore the potential of identifying modulatory compounds as therapeutic strategies targeting TRIM proteins. More specifically, TRIM protein activity can be regulated by inhibitors targeting any accessible functional domain. While clinical trials for TRIM-targeting drugs are still unavailable, preclinical studies have drawn attention to inhibitors acting on specific domains. For example, TRIM24 bromodomains can be effectively inhibited by compounds like Compound 34 and IACS-9571, emphasizing the promise of domain-specific approaches [192,193,194]. Regulation of TRIM protein levels by RNA has been observed in various cancers. In gastric cancer, circ_0091741 upregulates TRIM14 by sponging miR-330-3p, and miR-511 downregulates TRIM24, while miR-185 inhibits TRIM29 at transcriptional and protein levels. Additionally, lncRNA SDMGC regulates TRIM16 expression. In other cancers, for instance, TRIM11 is suppressed by miR-5193 in prostate cancer and by miR-24-3p in colon cancer [195,196]. In hepatocellular carcinoma, TRIM35 is downregulated by miR-4417 [197]. Additionally, in colon cancer, LINC00265 indirectly upregulates TRIM44 expression by inhibiting miR-216b-5p [198]. These findings suggest that developing miRNA-based therapies could provide a strategy to modulate TRIM protein expression in cancer. In addition, targeting TRIM proteins combined with immune checkpoint inhibitors has shown promising potential in enhancing antitumor immunity. Therapies that regulate TRIM protein activity may alleviate immunosuppression mediated by components in the tumor microenvironment, thereby improving the efficacy of PD-1/PD-L1 inhibitors [199]. From another perspective, to date, bortezomib (Velcade^®^) and carfilzomib (Kyprolis^®^) are two FDA-approved proteasome inhibitors used in the treatment of multiple myeloma [200]. Moreover, there are also some relevant clinical trials. For example, a recent Phase I clinical trial investigated the combination of TQB3602, a novel proteasome inhibitor, with AK105 (penpulimab), an anti-PD-1 monoclonal antibody, in patients with advanced cancers (NCT05333276). The above studies demonstrate that the UPS process can be effectively inhibited, suggesting the potential for future development of drugs targeting enzymes involved in UPS, including RING E3 ligases, such as TRIM proteins.

Moreover, TRIM proteins also have the potential to become diagnostic biomarkers. Future studies can focus on utilizing TRIM proteins for early gastric cancer diagnosis, for example, by detecting their expression in patient serum.

**Table 2:** Expression of TRIM proteins in gastric cancer and associated clinicopathological features.

Name	Expression in GC	Case Number	Prognostic Factors	Clinicopathological Features	Reference
TRIM3	High	40	OS	Gender	[83]
TRIM11	High	45	-	T stage, tumor size	[101]
		36	OS	T stage	[69]
		150	OS	Tumor size, depth of invasion, TNM stage, lymph node metastasis	[70]
TRIM14	High	117	OS	TNM stage, lymph node metastasis	[103]
TRIM15	Low	134	OS	Depth of invasion, TNM stage	[168]
	High	275	OS	Depth of invasion, tumor location, distant metastasis, lymph node metastasis, TNM stage	[151]
TRIM19	Low	1092	OS	SRC, tumor invasion, lymphatic invasion, pTNM stage	[170]
TRIM21	Low	64	OS	-	[174]
		90	OS	-	[137]
TRIM22	Low	90	OS	Tumor size, local invasion	[119]
TRIM23	High	81	OS	Tumor size, depth of invasion, TNM stage, tumor differentiation, lymph node metastasis, nerve invasion	[153]
TRIM24	High	133	OS	Tumor invasion, TNM stage, relapse	[105]
		90	OS	Depth of invasion, lymph node metastasis, TNM stage	[72]
TRIM25	High	90	OS	-	[118]
		378	OS	-	[178]
TRIM27	High	92	OS	Tumor size, depth of invasion	[149]
TRIM28	High	20	OS, FPS, PPS	-	[86]
		875	OS	-	[138]
		76	OS	Tumor size	[161]
		112	-	lymph node metastasis, TNM Stage	[162]
		91	OS	Peritoneal carcinomatosis	[163]
TRIM29	High	124	OS	Histological grade, tumor size, tumor stage, lymph node metastasis	[154]
		243	OS	Tumor stage, lymph node metastasis, tumor cell differentiation, TNM stage	[155]
		22	OS	-	[75]
		40	OS	Gender	[74]
	Low	80	OS	TNM stage, PD-L1, CD8	[179]
TRIM31	High	170	OS	TNM stage, depth of invasion, lymph node metastasis	[65]
		-	OS	T4 stage, N3 stage	[156]
TRIM32	High	142	OS, RFS	Lymphatic invasion	[157]
		61	OS	-	[78]
		876	OS	-	[109]
TRIM33	High	875	OS, FPS, PPS	-	[120]
TRIM37	High	120	OS	T stage, pTNM stage, lymph node metastasis	[164]
		124	OS, RFS	Gender, histological grade, venous invasion, lymphatic invasion, TNM classification, recurrence	[165]
TRIM44	High	112	OS	Macroscopic appearance, venous invasion, lymphatic invasion, recurrence	[158]
		40	OS	-	[80]
		75	OS	-	[81]
		384	OS, FPS, PPS	-	[181]
TRIM47	High	136	OS, DFS	Tumor differentiation, TNM stage	[145]
TRIM50	Low	124	OS	Tumor volume, M classification, pathological stage	[184]
TRIM54	High	384	OS	-	[167]
TRIM55	High	91	OS	T stage, TNM stage, lymph node metastasis	[159]
TRIM59	High	111	OS	T stage	[130]
		602	-	Smoking	[201]
	Low	631	OS	-	[147]
TRIM69	Low	162	-	Clinical stage I, metastasis, pathological grading G1–G2	[185]

List of abbreviations: OS (overall survival), FPS (first progression survival), PPS (post progression survival), DFS (disease-free survival), RFS (relapse-free survival), SRC (signet-ring cell carcinoma).

## 6. Conclusions

In conclusion, studies on TRIM proteins are gaining more and more attention. TRIM proteins have multiple functions in a variety of cellular processes in gastric cancer cells, including proliferation, apoptosis, metastasis, treatment resistance, and immune response, functioning as both oncogenic and tumor-suppressive factors. While a significant proportion of TRIM proteins act via the modulation of signaling pathways like Wnt/β-catenin, PI3K/AKT, and TGF-β/Smad, they also affect cellular activities through non-signaling mechanisms, such as regulation of gene transcription, as well as protein degradation and stability. Furthermore, TRIM protein expression in gastric cancer is associated with prognosis and clinicopathological features. These findings indicate TRIM proteins could be therapeutic targets and prognostic factors for this disease.

## Data Availability

No new data were created or analyzed in this study.
